# The incidence, risk factors, and clinical outcomes of acute kidney injury (staged using the RIFLE classification) associated with intravenous acyclovir administration

**DOI:** 10.1080/0886022X.2018.1487866

**Published:** 2018-12-27

**Authors:** Eun Ju Lee, Ha Nee Jang, Hyun Seop Cho, Eunjin Bae, Tae Won Lee, Se-Ho Chang, Dong Jun Park

**Affiliations:** a Department of Internal Medicine, Gyeongsang National University Hospital, Jinju, South Korea;; b Department of Internal Medicine, Changwon Gyeongsang National University Hospital, Changwon, South Korea;; c Department of Internal Medicine, College of Medicine, Gyeongsang National University, Jinju, South Korea;; d Institute of Health Science, Gyeongsang National University, Jinju, South Korea

**Keywords:** Acute kidney injury, acyclovir, infection, prevention, risk factor

## Abstract

Intravenous (IV) acyclovir is commonly administered medication for viral infection but is well known for its nephrotoxicity. However, there was no study for incidence, risk factors, and clinical outcomes of acute kidney injury (AKI) associated with IV acyclovir administration. We retrospectively reviewed the medical records of 287 patients who were medicated IV acyclovir from January 2008 to May 2013 in Gyeongsang National University Hospital. All had documented medical histories and underwent medical review. Demographic data, risk factors, concomitant drugs, laboratory findings and outcome were gathered from the medical records and analyzed. AKI occurred in 51 patients (17.8%). As per RIFLE classification, renal injury was graded as either at risk of renal dysfunction (62.7%), renal injury (15.6%), and renal failure (21.6%). There was no significant difference in age, sex, total dose, drug duration, and presence of hydration between AKI and non-AKI group. However, systolic pressure, underlying diabetes, concomitant vancomycin and non-steroidal anti-inflammatory drugs (NSAIDs) use was positively correlated with AKI occurrence (*p* = .04, *p* < .001, 0.01, and 0.04, respectively). Two patients underwent hemodialysis and these patients died. Higher mortality was observed in AKI patients (*p* < .001). Multivariate analysis also presented that presence of diabetes, concomitant NSAIDs, and vancomycin use was independent risk factor of acyclovir associated with AKI (*p* = .001, OR 3.611 (CI: 1.708–7.633), *p* = .050, OR 2.630 (CI: 1.000–6.917), and *p* = .009, OR 4.349 (CI: 1.452–13.022), respectively). AKI is relatively common in patients administrating acyclovir injection. Physicians should attempt to prevent, detect, and manage acyclovir associated AKI in patients prescribing acyclovir due to possible association of poor prognosis.

## Introduction

Acyclovir is widely used to treat herpes virus infections, particularly those associated with herpes simplex virus and varicella-zoster virus. Randomized controlled trials have shown that acyclovir effectively reduces the duration and severity of painful lesions, prevents complications, and decreases recurrence [1–4]. Acyclovir is remarkably well-tolerated in most patients [4]. However, several severe toxic reactions have been reported. Acute kidney injury (AKI) (precipitation of relatively insoluble acyclovir crystals in the renal tubules) is an occasional complication of intravenous (IV) therapy [5,6] In one small study, 11 of 23 (48%) outpatients with herpes zoster treated via IV acyclovir developed AKI, typically within 2–3 days of infusion (median increase in serum creatinine level, 0.8 mg/dL; range: 0.3–3.7 mg/dL) [7]. The adverse effects of IV acyclovir (which are sometimes fatal) are now mentioned in drug-prescribing manuals [8]. Although older patients were reported to be at risk of AKI caused by oral acyclovir [9], no large study has explored whether AKI is associated with the use of IV acyclovir. Therefore, we performed this retrospective study to evaluate the incidence, risk factors, and clinical outcomes of patients with acyclovir-associated AKI staged using the RIFLE classification [10]. We hypothesized that the patient’s demographic, clinical, laboratory characteristics, and concomitant drugs would affect the incidence, risk factors, and clinical outcomes.

## Methods

We retrospectively reviewed the medical records of 287 patients who received IV acyclovir at Gyeongsang National University Hospital from January 2008 to May 2013. All had extensive medical histories and underwent both a medical review and a general physical examination. We collected demographic data (sex, age, weight, height); information on comorbidities (diabetes mellitus [DM], hypertension, chronic kidney disease [CKD], malignancy); the most recent blood pressure (BP) before IV acyclovir administration (BP); data on fluid management when taking acyclovir; the total dose and duration of acyclovir treatment; data on concomitant medications (non-steroidal anti-inflammatory drugs [NSAIDs], mannitol, diuretics, ceftriaxone, angiotensin-converting enzyme inhibitors [ACEIs], and angiotensin receptor blockers [ARBs]); information on the use of contrast dye; and all other biochemical data collected during treatment. Our exclusion criteria were: (1) age <18 years; (2) lack of follow-up laboratory data; (3) any dialysis in the prior 120 days (to exclude patients with end-stage renal disease); (4) initial shock; and, (5) acute renal failure that developed prior to acyclovir treatment.

CKD was defined as disease associated with an estimated glomerular filtration rate (eGFR) < 60 mL/min/1.73 m^2^ using the formula of the Modification of Diet in Renal Disease (MDRD) study (1.86 × serum creatinine level) − 1.154 × (age) − 0.203) × (0.74 if female) × (1.210 if black). Creatinine level was measured using the Jaffe method. A need for hydration was reflected by IV fluid administration at 1 mL/kg/h during acyclovir infusion. AKI was defined and staged using the RIFLE system.^10^ We used the serum creatinine level and the eGFR to define RIFLE categories because we lacked data on the 6- and 12-h urine volumes. If the baseline creatinine values were unknown, the serum creatinine level was estimated using the MDRD formula assuming that the eGFR was 75 mL/min/1.73 m^2^ (the normal value). The study protocol was approved by the Institutional Review Board of Gyeongsang National University Hospital (IRB no: 2014–08-004).

All continuous variables are expressed as means ± standard deviation (SD). We used Pearson’s chi-squared test to compare qualitative differences. Fisher’s exact test was automatically applied if the number of expected cases was less than five. The significance of differences in continuous variables was explored using the independent samples t-test. We employed multivariate logistic regression to identify significant risk factors for AKI development among the factors identified via simple regression. All statistical analyses were performed using SPSS for Windows software (ver. 16.0). A *p* values < .05 was taken to indicate statistical significance.

## Results

### Clinical and laboratory findings

Table 1 summarizes the demographic data, underlying diseases, duration of acyclovir therapy and total dose, other nephrotoxic drugs taken, and laboratory data of the 287 patients on IV acyclovir. The average patient age was 55.3 years and 56.8% were male. In total, 67 patients (23%) had underlying hypertension, of whom 13 took an ACEI or an ARB (4.5%). Only 12 patients had underlying CKD. A total of 44 patients had DM (15.3%) and 105 had cancer (36.6%). Concomitant drugs were given to some patients (a radiocontrast dye [21.3%], ceftriaxone [15.3%], vancomycin [5.6%], NSAIDs [8.4%], mannitol [2.8%], and diuretics [3.1%]).

**Table 1.** Baseline clinical characteristics and laboratory findings of patients.

Age (yr)	55.3 ± 17.8
Male (%)	163 (56.8)
Body weight (kg)	61.0 ± 11.8
Height (cm)	162. 9 ± 10.1
Systolic pressure (mmHg)	119.2 ± 16.2
Diastolic pressure (mmHg)	75.2 ± 11.0
Drug duration (days)	8.8 ± 5.6
Total drug doses (mg)	12527.7 ± 9096.1
Hydration (%)	149 (51.9%)
Hydration amount (ml/hr)	63.1 ± 31.8
Kinds of infusion fluid	
0.45% saline	36 (22.2%)
0.90% saline	113 (77.8%)
Underlying disease	
DM	44 (15.3%)
Hypertension	66 (23%)
CKD	12 (4.2%)
Cancer	105 (36.6%)
Concomitant drugs	
Radiocontrast dye	61 (21.3%)
Ceftriaxone	44 (15.3%)
ARB or ACEi	13 (4.5%)
Vancomycin	16 (5.6%)
NSAIDs	24 (8.4%)
Mannitol	8 (2.8%)
Diuretics	9 (3.1%)
Laboratory findings	
WBC (×10^3^/*u*L)	7.8 ± 3.4
Hemoglobin (g/dL)	12.0 ± 2.2
Platelet (×10^3^ /mm^3^)	165.3 ± 54.5
Sodium (mmol/L)	138.6 ± 7.8
Potassium (mmol/L)	4.6 ± 1.4
Albumin (g/dL)	3.6 ± 0.7
BUN (mg/dL)	16.8 ± 11.6
Cr (mg/dL)	0.9 ± 0.7
CRP (mg/L)	24.2 ± 7.4

ARB: Angiotensin Receptor Blocker; ACEi: Angiotensin Converting Enzyme Inhibitor; WBC: White Blood Cell; NSAIDs: Non-Steroidal Anti-inflammatory Drugs; CRP: C-reactive Protein; BUN: Blood Urea Nitrogen; Cr: Creatinine; DM: Diabetes Mellitus; CKD: Chronic Kidney Disease.

### The incidence rates of AKI and differences between the AKI and non-AKI groups

**Figure 1.**
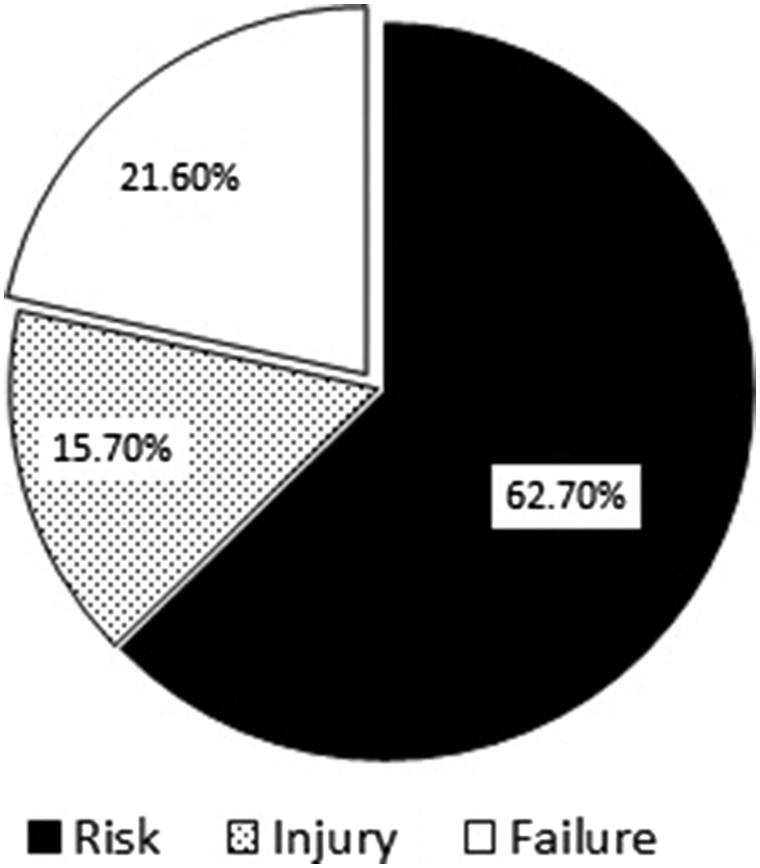
AKI categorization according to RIFLE classification.

AKI developed in 51 patients (17.8%), of whom 32 were in the ‘Risk’ category (62.7%), 8 were in the ‘Injury’ category (15.6%), and 11 were in the ‘Failure’ category (21.6%) (Figure 1). No patient was in the ‘Loss’ category or progressed to end-stage renal disease. We found no significant difference in terms of age, sex, total drug dose, treatment duration, the need for hydration, hydration amount, or kinds of infusion fluid between the AKI and non-AKI group, but body weight, height, and systolic blood pressure (SBP) were significantly higher in the AKI group. Also, AKI developed significantly more often in DM patients (*p* < .001) (Table 2). It is well-known that underlying CKD is an important risk factor for AKI. In total, 12 patients (4.2%) had underlying CKD, but this did not affect the development of AKI (*p* = .48). Concomitant medications (radiocontrast dye, ceftriaxone, ACEIs or ARBs, mannitol or diuretics) did not affect the AKI frequency (*p* = .27, 1.00, 0.48, 0.64, and 0.66, respectively). However, concomitant vancomycin or NSAIDs use increased the frequency of AKI (*p* = .01 and .04, respectively) (Table 2). The white blood cell count and the levels of hemoglobin, platelets, electrolytes (sodium and potassium), and C-reactive protein (CRP) did not differ between the AKI and non-AKI groups. DM, and concomitant NSAID and vancomycin use, were independent risk factors for acyclovir-associated AKI in both univariate and multivariate analyses (Table 3).

**Table 2.** Clinical and laboratory data of the AKI group and non-AKI group.

	AKI group (*n* = 51)	Non-AKI group (*n* = 236)	*p* Value
Age (yr)	55.7 ± 15	55.2 ± 18.2	.85
Male (%)	33 (64.7%)	153 (55.1%)	.22
Body weight (kg)	65.0 ± 10.8	60.1 ± 11.8	.02
Height (cm)	166.8 ± 7.6	162.1 ± 10.4	.02
Systolic pressure (mmHg)	123.5 ± 18.7	118.3 ± 15.5	.04
Diastolic pressure (mmHg)	76.0 ± 9.7	75.1 ± 11.3	.58
Drug duration (days)	9.1 ± 6.9	8.7 ± 5.3	.59
Total drug doses (mg)	14413.1 ± 10689.4	12116.8 ± 8634.1	.10
Hydration (%)	27 (52.9%)	122 (51.6%)	1.00
Hydration amount (ml/hr)	66.7 ± 35.3	62.4 ± 31.1	.38
Kinds of infusion fluid			.63
0.45% saline	6 (22.2%)	30 (25.0%)	
0.90% saline	21 (77.8%)	92 (75.0%)	
Underlying disease			
DM	16 (31.4%)	28 (11.9%)	.002
Hypertension	14 (27.5%)	52 (22%)	.46
CKD	1 (2.0%)	11 (4.7%)	.48
Cancer	17 (33.3%)	88 (37.3%)	.68
Concomitant drugs			
Radiocontrast dye	9 (17.6%)	52 (22.0%)	.27
Ceftriaxone	8 (15.7%)	36 (15.3%)	1.00
ARB or ACEi	1 (2.0%)	12 (5.1%)	.48
Vancomycin	7 (13.7%)	9 (3.8%)	.01
NSAIDs	8 (15.7%)	16 (6.8%)	.04
Mannitol	2 (3.9%)	6 (2.5%)	.64
Diuretics	2 (3.9%)	7 (3.0%)	.66
Laboratory findings			
WBC (×10^3^/*u*L)	8.0 ± 2.6	7.6 ± 4.2	.15
Hemoglobin (g/dL)	12.3 ± 2.2	11.9 ± 2.2	.25
Platelet (×10^3^ /mm^3^)	152.2 ± 86.4	178.4 ± 22.6	.53
Sodium (mmol/L)	135.0 ± 19.6	136.7 ± 10.2	.37
Potassium (mmol/L)	4.7 ± 2.0	4.5 ± 0.8	.34
Albumin (g/dL)	3.6 ± 0.9	3.6 ± 0.7	.99
BUN (mg/dL)	19.5 ± 14.9	16.2 ± 10.7	.07
Creatinine (mg/dL)	0.8 ± 0.4	0.9 ± 0.8	.62
CRP (mg/L)	27.1 ± 11.5	21.3 ± 3.2	.27
RIFLE classification			
Risk	32 (62.7%)		
Injury	8 (15.6%)		
Failure	11 (21.6%)		
Dialysis	2 (3.92%)		
Death	8 (15.7%)	8 (3.4%)	.002

ARB: Angiotensin Receptor Blocker; ACEi: Angiotensin Converting Enzyme Inhibitor; WBC: White Blood Cell; NSAIDs: Non-Steroidal Anti-Inflammatory Drugs; CRP: C-reactive Protein; BUN: Blood Urea Nitrogen; Cr: Creatinine; DM: Diabetes Mellitus; CKD: Chronic Kidney Disease.

**Table 3.** Independent risk factors for the development of AKI.

Variables	Univariate analysis			Multivariate analysis		
	*p* value	OR	95% CI	*p* value	OR	95% CI
SBP		NA	NA	151	1.014	0.995–1.003
DM	<.001	3.620	1.793–7.309	.001	3.611	1.708–7.633
NSAIDs use	.048	2.422	1.010–6.002	.050	2.630	1.000–6.917
Vancomycin use	.015	3.804	1.348–10.740	.009	4.349	1.452–13.022

SBP: Systolic Blood Pressure; DM: Diabetes Mellitus; NSAIDs: Non-Steroidal Anti-Inflammatory Drugs; NA: Not Applicable; OR: Odds Ratio; CI: Confidence Interval.

### Outcomes of acute kidney injury

Ultimately, 16 of the 287 (5.57%) patients died; most patients recovered from AKI although renal replacement therapy was required by 2 patients, who were in the ‘Failure’ category and died. The mortality rate was significantly higher in the AKI group: eight patients (15.7%) compared to eight in the non-AKI group (3.4%) (*p* < .001). In terms of the RIFLE classification, there were five deaths (15.6%) among patients in the ‘Risk’ category, one (12.5%) among those in the ‘Injury’ category, and two (18.2%) among those in the ‘Failure’ category. No AKI patient developed end-stage renal disease requiring maintenance hemodialysis after the 3-month follow-up.

## Discussion

We found that the incidence rate of AKI associated with use of IV acyclovir was 17.8%. Using the RIFLE terms, AKI was associated with ‘risk’ (62.7%), ‘injury’ (15.6%), and ‘failure’ (21.7%). AKI was more frequent in patients with DM and those who took concomitant NSAIDs or vancomycin. Mortality was higher in AKI than non-AKI patients, although most patients recovered from AKI.

It has been relatively well-documented that IV acyclovir is associated with AKI, in 12–48% of patients [11]. The risk factors for AKI include rapid infusion of high-dose acyclovir, volume depletion, and underlying renal impairment. In the present study, the incidence of AKI was 17.8%. Neither treatment duration nor total drug dose significantly affected the AKI rate. Also, underlying renal impairment was not an important risk factor for AKI development, although the number of CKD patients was small. Slow acyclovir infusion, hydration following our institutional policy, and dose adjustment depending on renal function may explain the relatively low incidence of AKI in this study. Prevention of acyclovir-induced AKI is aided by avoiding rapid bolus drug infusions, IV saline injection to replete volume and maintain urinary flow, use of the lowest possible drug dose, and dose adjustment in patients exhibiting renal impairment. An advantage of our study was that we enrolled a high number of patients treated drawn from a single center [7,12,13]

The proposed mechanism of acyclovir-associated renal injury is drug precipitation and crystallization in the renal tubules, which obstructs the tubules and possibly causes necrosis [14,15]. Typically, acyclovir is rapidly excreted in the urine. AKI usually develops (if at all) within 2–3 days after IV acyclovir infusion [11]. However, any renal insufficiency is mostly asymptomatic although it may be associated with nausea, vomiting, a decreased urine output, and flank or abdominal pain within 24–48 h of acyclovir infusion. Therefore, a renal function test should be performed 48–72 h after drug infusion commences.

Drug-induced AKI is seen in 8–60% of all cases of in-hospital AKI and is a recognized source of significant morbidity and mortality [16]. We found that mortality was higher in AKI patients taking IV acyclovir, although the cause of death was principally underlying diseases such as cancer. It is well-known that the renal prognosis of patients with acyclovir-associated AKI is usually excellent if intervention is timely [11]. Most of our patients recovered from AKI. Treatments for overt acute renal failure (ARF) caused by acyclovir include drug withdrawal, diuresis using loop diuretics, adjustment of medication dose, a restricted diet, metabolic and volume management, and dialysis to allow for metabolic and volumetric control and removal of acyclovir.

Other reports have found that combination therapy with NSAIDs was an independent risk factor for the development of antibiotic-induced nephrotoxicity [17]. Also, triple therapies with diuretics, ACIs or ACBs, and NSAIDs were associated with an increased AKI risk [18]. It is well- known that vancomycin is an independent risk factor for AKI development. Vancomycin increases the level of reactive oxygen and triggers changes in mitochondrial function that in turn induce proliferation of renal proximal tubule epithelial cells [19], ultimately increasing the risk of AKI. We also found that concomitant use of NSAIDs and vancomycin was an independent risk factor for the development of acyclovir-associated AKI in both univariate and multivariate analyses. DM is the single largest risk factor for AKI, which may in turn trigger CKD [20]. Many studies have shown that DM is a risk factor for AKI development in association with nephrotoxic drug use [20,21]. We also found that DM was a significant independent risk factor for AKI development in those taking acyclovir.

Two reports showed that additional administration of ceftriaxone was risk factor of IV acyclovir associated AKI [22,23]. Ceftriaxone alone injection has not been known to cause nephrotoxicity although low molecular weight proteinuria has been reported during treatment [24]. Vomiero et al. found that there was a significant increase in serum creatinine after combination therapy ceftriaxone and IV acyclovir in 70% children patients and the degree of renal impairment significantly correlated with acyclovir dose [22]. Rao et al. revealed that ceftriaxone was significant risk factor for ‘failure’ category in children patients administrating IV acyclovir through a matched case control study [23]. Unlike the previous two studies, our studies did not show the same results. It is assumed that the reason for the difference might be associated with different patient type, adults and children, and definition of renal dysfunction. Although not the main purpose of this study, combination therapy ceftriaxone and IV acyclovir did not increase the risk of AKI in adults. Further well-designed study remains to be elucidated to confirm the association.

Our study had several limitations. First, the work was retrospective in nature, possibly compromising data accuracy; as we relied on medical records, we were unable to identify other possible causes of AKI. Second, although we used the RIFLE system to stage AKI, only the serum creatinine level and the eGFR were used as inputs to the MDRD equation; we lacked data on urine output (which was not routinely recorded). Therefore, the incidence and severity of AKI may have been underestimated. Third, the research was conducted at a single center, and the results may thus not be generalizable. A well-designed, randomized prospective study is required to confirm our results. However, we believe that these limitations may have been offset by our large sample size. The quality of the laboratory data was high because the patients were hospitalized, and all assays were performed in the same laboratory. We excluded patients for whom follow-up laboratory data were missing; this may have affected our results.

In conclusion, AKI is relatively common in patients receiving IV acyclovir. Physicians should seek to prevent, detect, and manage acyclovir-associated AKI, especially in patients taking concomitant NSAIDs and vancomycin, and those with DM, who may have poor prognoses if AKI develops.
